# Juvenile idiopathic arthritis is associated with potentially pathogenic glycosylation of IgG

**DOI:** 10.1186/1546-0096-10-S1-A5

**Published:** 2012-07-13

**Authors:** Altan Ercan, Melissa Hazen, Mary Beth Son, Susan Kim, Fatma Dedeoglu, Robert P Sundel, Robert C Fuhlbrigge, Jing Cui, Nancy A Shadick, Michael E Weinblatt, Michael Spigarelli, David N Glass, Susan D Thompson, Peter A Nigrovic

**Affiliations:** 1 Brigham and Women's Hospital, Boston, MA, USA; 2 Brigham and Women's Hospital, Children's Hospital Boston, Boston, MA, USA; 3 Children's Hospital Boston, Boston, MA, USA; 4 Cincinnati Children's Hospital Medical Center, Cincinnati, OH, USA

## Purpose

The structure of human IgG includes 2 internal glycans that modulate the conformation and function of the Fc region. Adult-type rheumatoid arthritis (RA) has been associated with an elevated proportion of hypogalactosylated (G0) IgG that exhibits enhanced fixation of complement and greater pathogenicity in experimental murine arthritis. We aimed to determine the profile of IgG glycans in normal children and in those with juvenile idiopathic arthritis (JIA), for comparison with healthy adults and adult RA controls.

## Methods

Serum samples were collected from healthy children, children with JIA naïve to disease-modifying anti-rheumatic therapy, healthy adults, and adults with RA. Using a validated high-throughput method, glycans were liberated enzymatically from whole serum and identified by high-pressure liquid chromatography (HPLC). The G0 fraction, reflecting largely the IgG pool, was normalized to monogalactosylated (G1) IgG, which is constant across the age spectrum. Results were correlated with clinical and laboratory phenotype.

## Results

Samples were analyzed from 112 healthy children, 40 children with JIA, 247 healthy adults and 204 adults with RA. Among normal controls, the proportion of IgG that is G0 (expressed as G0/G1 ratio) was found to describe a smooth U-shaped curve across the age spectrum. The G0/G1 ratio nadirs between late adolescence and age 40 years at approximately 0.8, while it increases to 1.1 in early childhood and 1.3 in later life. We confirmed the known association of RA with an aberrant increase in G0 fraction, in particular among patients positive for anti-cyclic citrullinated peptide (CCP) antibodies. Children with JIA demonstrated striking aberrancy in G0/G1 ratio, suggesting a shared pathogenic mechanism with adult arthritis. Analysis of G0/G1 by JIA phenotype, including from an additional 100 JIA samples and matched controls, is anticipated within the next several months.

## Conclusion

Our results define the normal pattern of IgG glycosylation across the age spectrum and highlight an increase in G0/G1 ratio in early childhood that may impact normal pediatric immune function and contribute to the tendency of JIA to affect younger children.

## Disclosure

Altan Ercan: None; Melissa Hazen: None; Mary Beth Son: None; Susan Kim: None; Fatma Dedeoglu: None; Robert P. Sundel: None; Robert C. Fuhlbrigge: None; Jing Cui: None; Nancy A. Shadick: Abbott Laboratories, 2, Amgen Inc., 2, Biogen Idec, 2, Crescendo Biosciences, 2; Michael E. Weinblatt: Biogen Idec, 2, 5, Crescendo Bioscience, 2, 5; Michael Spigarelli: None; David N. Glass: None; Susan D. Thompson: None; Peter A. Nigrovic: None.

